# Whole Plant Temperature Manipulation Affects Flavonoid Metabolism and the Transcriptome of Grapevine Berries

**DOI:** 10.3389/fpls.2017.00929

**Published:** 2017-06-06

**Authors:** Chiara Pastore, Silvia Dal Santo, Sara Zenoni, Nushin Movahed, Gianluca Allegro, Gabriele Valentini, Ilaria Filippetti, Giovanni Battista Tornielli

**Affiliations:** ^1^Department of Agricultural Sciences, University of BolognaBologna, Italy; ^2^Department of Biotechnology, University of VeronaVerona, Italy

**Keywords:** grape, ripening, flavonoid, transcriptome, temperature

## Abstract

Among environmental factors, temperature is the one that poses serious threats to viticulture in the present and future scenarios of global climate change. In this work, we evaluated the effects on berry ripening of two thermal regimes, imposed from veraison to harvest. Potted vines were grown in two air-conditioned greenhouses with High Temperature (HT) and Low Temperature (LT) regimes characterized by 26 and 21°C as average and 42 and 35°C as maximum air daily temperature, respectively. We conducted analyses of the main berry compositional parameters, berry skin flavonoids and berry skin transcriptome on HT and LT berries sampled during ripening. The two thermal conditions strongly differentiated the berries. HT regime increased sugar accumulation at the beginning of ripening, but not at harvest, when HT treatment contributed to a slight total acidity reduction and pH increase. Conversely, growing temperatures greatly impacted on anthocyanin and flavonol concentrations, which resulted as strongly reduced, while no effects were found on skin tannins accumulation. Berry transcriptome was analyzed with several approaches in order to identify genes with different expression profile in berries ripened under HT or LT conditions. The analysis of whole transcriptome showed that the main differences emerging from this approach appeared to be more due to a shift in the ripening process, rather than to a strong rearrangement at transcriptional level, revealing that the LT temperature regime could delay berry ripening, at least in the early stages. Moreover, the results of the in-depth screening of genes differentially expressed in HT and LT did not highlight differences in the expression of transcripts involved in the biosynthesis of flavonoids (with the exception of PAL and STS) despite the enzymatic activities of PALs and UFGT being significantly higher in LT than HT. This suggests only a partial correlation between molecular and biochemical data in our conditions and the putative existence of post-transcriptional and post-translational mechanisms playing significant roles in the regulation of flavonoid metabolic pathways and in particular of anthocyanins.

## Introduction

Agriculture, and in particular viticulture, is highly dependent upon climatic conditions during the growing season. The predicted climate change therefore presents a major challenge for wine production. Although *Vitis vinifera* shows large variations in terms of tolerance to abiotic summer stresses, i.e., high temperature (HT) and radiation and low water availability (Palliotti and Poni, 2016), the effects of a temperature increase on berry composition have been widely studied, in terms of both extreme heatwaves or mild-to-moderate increase during ripening. Since berry temperature and solar radiation often act synergistically and sun exposure of grape bunches can be modified by viticulture practices, several researches have focused on the effect of both parameters simultaneously (Bergqvist et al., 2001; Spayd et al., 2002; Downey et al., 2004; Cortell and Kennedy, 2006; Tarara et al., 2008; Azuma et al., 2012; Movahed et al., 2016) and only a few computed the precise role of temperature in these multi-factor studies (Mori et al., 2005, 2007; Yamane et al., 2006; Cohen et al., 2012; Sadras et al., 2013a,b; Rienth et al., 2016).

High sugar concentration at harvest is often associated with thermal increase, as indicated by the trend observed in the last decades (Petrie and Sadras, 2008; Mira de Orduña, 2010; Sadras and Petrie, 2011). However, several experiments showed that sugar accumulation is not or only slightly affected (Spayd et al., 2002; Mori et al., 2005; Mori et al., 2007; Sadras and Denison, 2009; Movahed et al., 2016), or sometimes even reduced (Greer and Weston, 2010; Greer et al., 2010; Carbonell-Bejerano et al., 2014; Rienth et al., 2016) by air temperature increase. The different results can probably be ascribed to variation in diurnal temperature levels, since temperatures over 30°C may lead to the stopping of soluble solids transport from leaves to berry, but may sometimes indirectly cause a higher concentration by evaporative loss (Keller, 2010).

Temperature has been known for some time to have significant effects on berry acidity, accelerating the breakdown of malic acid (Rienth et al., 2016) and decreasing the titratable acidity the greater the heat summation (Tarara et al., 2008). Intriguingly, other studies suggested a cultivar-dependent thermal response of acidity and pH (Bergqvist et al., 2001; Sadras et al., 2013a,b; Movahed et al., 2016).

Of particular interest are the effects of temperature on the phenylpropanoid biosynthetic pathway, involved in the biosynthesis of flavonoids (anthocyanins, flavonols, and tannins) that play a crucial role in grape and wine composition with regards to color, bitterness and stability, and also in the biosynthesis of non-flavonoid compounds (i.e., stilbenes). A negative correlation between elevated temperature during the day (over 30°C) and anthocyanin concentrations has recently been explored (Mori et al., 2007; Carbonell-Bejerano et al., 2013; Movahed et al., 2016). Some authors pointed out the effect of increasing temperatures on the reduction of the enzymatic activity of some key enzymes involved in flavonoid biosynthesis as phenylalanine ammonia-lyase (PAL), which presides the first step of general phenylpropanoid biosynthesis, and UDP-glucose:flavonoid 3-O-glucosyltransferase (UFGT), which is involved in the last and specific step of anthocyanin biosynthesis (Mori et al., 2007; Movahed et al., 2016).

Flavonols are known to behave as UV-protectants and to play a role in co-pigmentation with anthocyanins. Flavonols in the berry can be affected by sunlight exposure, which usually promotes strong enhancement in concentrations and in the expression of flavonol biosynthesis-related genes (Spayd et al., 2002; Downey et al., 2004; Czemmel et al., 2009). On the contrary, temperature seems to have less effect than light in flavonol synthesis control and under thermal increase flavonols can be unaffected (Tarara et al., 2008) or slightly reduced (Azuma et al., 2012). Temperature appears to have little impact on tannins (Cohen et al., 2012) whose accumulation in skins and seeds occurs predominantly before veraison (Downey et al., 2004).

Grapevine transcriptomic analysis has provided a wealth of data concerning the mechanisms responsible for the temperature effects on berry composition, especially on sugars, acidity and anthocyanin concentrations (Carbonell-Bejerano et al., 2013; Pastore et al., 2013; Rienth et al., 2014, 2016; Lecourieux et al., 2017). Several authors reported that the loss of anthocyanin synthesis following HT is due to the reduced expression of anthocyanin biosynthetic genes (Yamane et al., 2006; Azuma et al., 2012; Lecourieux et al., 2017). Sometimes, however, despite a sharp reduction in terms of anthocyanin concentration, a concomitant reduction in anthocyanin biosynthetic genes expression was not found, as reported for Muscat Hamburg berries on fruiting cuttings (Carbonell-Bejerano et al., 2013) and in Cabernet Sauvignon and Sangiovese vines grown under increasing temperature (Mori et al., 2007; Movahed et al., 2016). In these cases, also an involvement of anthocyanin degradation, implying the action of peroxidases should be hypothesized (Mori et al., 2007; Movahed et al., 2016) as it was previously seen in other plant species, as *Brunfelsia* flower petals (Vaknin et al., 2005), litchi (Zhang et al., 2005), and strawberry fruits (Chisari et al., 2007).

Despite recent progress, the direct and indirect effects of temperature on the grape ripening process, and specifically on flavonoid composition, are far from being completely unraveled. In particular, there is growing interest in charting the impact of temperature in specific viticultural areas and different seasons on flavonoid composition. Here, we analyzed the grapevine cultivar Sangiovese, the most cultivated Italian variety, comparing the effects of two thermal regimes on the berry skin biochemical composition, flavonoid-related enzymatic activity, and whole transcriptome during ripening.

## Materials and Methods

### Grapevine Plant Material and Growing Conditions

Experiments on grapevine berries were conducted in 2012 on 6-year-old uniformly potted plants (*V. vinifera* cv. Sangiovese). The vines, grafted on SO4 rootstocks, were grown in 30-liter pots containing a 1:1 mixture of sand and soil (27% sand, 46% silt and 27% clay, clay loam soil). The number of shoots was standardized to nine per vine. In addition, to achieve a uniform leaf area on all the vines, the tip of each shoot was removed and 15 main leaves were maintained before the experiment started. At the beginning of bunch closure [BBCH 77, (Lorenz et al., 1995)], 10 vines were selected and bunch numbers were adjusted to 11–12 per vine.

The vines were assigned to two treatments: low temperature (LT) and HT. Five LT vines were placed from 1 week before veraison to harvest in a plastic greenhouse (20 m^3^) where the air temperature was controlled by a cooler and a fan was used to homogenize environmental conditions in the greenhouse (Supplementary Figure 1). During the night, the tunnel was opened.

Five HT vines were placed in an identical plastic greenhouse, without fan, whose basal segment was open but all the canopies were covered to maintain similar illumination to the LT vines.

The average, maximum and minimum air temperatures were recorded using air temperature sensors (TL20, 3M, Milan, Italy) in both greenhouses during the ripening period (**Table 1**).

**Table 1:** Air temperature measurements.

Treatment	Average air temperature (°C)	Maximum air temperature (°C)	Minimum air temperature (°C)
HT	26.4	41.7	11.8
LT	21.3	35.0	10.1


*Average, maximum, and minimum air temperature recorded during the experiment under the two different growing regimes. LT = low temperature, HT = high temperature.*

Both greenhouses were made of polyethylene film (MOP, Bologna, Italy) that did not alter the spectral composition of light. The incident light during the day, which outside ranged from 500 to 2000 μmol⋅m^2^⋅s^-1^, was reduced by the polyethylene film up to 12% within the visible range. The humidity recorded during the experiment was comparable between LT and HT greenhouses.

All vines were automatically watered daily and were well supplied with nutrients.

### Berry Temperature Monitoring

Berry temperature was monitored in 10 bunches from each treatment using 10 T-type thermocouples (RS component, Milan, Italy) positioned in the sub-cuticular tissues of the berry skin. Each probe was then connected to a CR10X data logger (Campbell Scientific Ltd, Leicestershire, United Kingdom), registering temperature data every 20 min during the development period.

### Berries Sampling

Berries were sampled before the treatment (T0, 1 week before veraison), at veraison (T1) and 10 (T2), 20 (T3), 32 (T4), and 45 (T5) days after veraison, corresponding to harvest. The berries were collected at the same time of day (9–10 am). At T0 five berries from each of the ten vines were sampled and pooled and this procedure was repeated 4 times to create four independent biological replicates of 50 berries each. Upon thermal treatment imposition, nine berries were randomly selected from each of the five treated vines and pooled. The same sampling procedure was repeated four times to create four independent pools of 45 berries per each sampling date/treatment combination. In total, the experiment entailed the collection and the analysis of 44 berry samples [4 control samples + (2 thermal regimes × 5 stages × 4 biological replicates)].

From each biological replicate about 20 berries were weighed and directly tested for the evaluation of soluble solids (°Brix), titratable acidity and pH. The remaining berries were peeled and the skins were immediately frozen in liquid nitrogen and stored at -80°C for subsequent metabolic analyses, enzyme activity and expression analysis.

### Soluble Solids, Titratable Acidity, and pH Measurements

The sampled berries were crushed and the must was sieved and used for soluble solids analysis with a temperature-compensating CR50 refractometer (Maselli Misure Spa, Parma, Italy). We then diluted 5 ml of the same must seven times with bi-distilled water for titration using a Crison Compact Titrator (Crison, Barcelona, Spain) with 1 N, 0.5 N or 0.25 N NaOH (Sigma-Aldrich, St. Louis, MO, United States), according to the stage of berry ripening to obtain pH and titratable acidity data (expressed as g L^-1^ of tartaric acid equivalents).

### Analysis of Grape Berry Anthocyanins and Flavonols

Total anthocyanins and flavonols were analyzed in all 44 samples by soaking 2–3 grams of peeled skins, depending on the berry phenological stage, per each sampling date/treatment combination in 50 mL methanol for 24 h (Mattivi et al., 2006), then storing the extracts at -20°C.

To analyze the total concentrations of each flavonol aglycone, an aliquote of 5 ml of methanolic extract was completely dried under vacuum. To achieved the acid hydrolization of flavonol glucosides, the pellet was resuspended in 2.5 ml of methanol and 2.5 ml of 2M trifluoroacetic acid (Sigma–Aldric, Saint Louis, MO, United States) in milliQ water. The reaction was conducted at 100°C in a boiling hot water bath, with a condenser, for 2 h. The reactions product was then completely dried under vacuum and the pellet obtained resuspended in 1 ml of methanol until HPLC analyses (Mattivi et al., 2006).

HPLC separation and quantifications of anthocyanins and flavonols (Mattivi et al., 2006) were performed on a Waters 1525 HPLC (Waters, Milford, MA, United States) equipped with a diode array detector (DAD) and a Phenomenex (Castel Maggiore, Bologna, Italy) reversed-phase column (RP18, 250 mm × 4 mm, 5 μM). Anthocyanins were quantified at 520 nm using an external calibration curve with malvidin-3-glucoside chloride as the standard (Sigma-Aldrich). Flavonols were quantified at 370 nm with the corresponding external standards (myricetin, quercetin, and kaempferol) purchased from Extrasynthese (Genay, France).

### Analysis of Skin Berry Tannins

Skin tannins extraction was performed following the procedure proposed by Downey et al. (2003): About 100 mg of skins per each sampling date/treatment combination were ground to a fine powder separately, extracted with a solution containing 70% acetone for 24 h in dark room and measured by HPLC using the same equipment used for anthocyanins analysis. After free monomers were removed, the tannin content was determined by acid-catalyzed cleavage in the presence of excess phloroglucinol as described by Kennedy and Jones (2001). Individual reversed-phase HPLC separations were used to determine the abundance of free monomers and cleaved proanthocyanidins by measuring absorbance at 280 nm (Downey et al., 2003). The concentrations of free monomers and hydrolyzed terminal subunits were determined from standard curves prepared with commercial standards of catechin, epicatechin, epicatechin-gallate and epigallocatechin (Extrasynthese, France).

### Enzymatic Activity Assays

Berry skins (0.2 gr) were ground with a mortar and pestle in liquid nitrogen to a fine powder. For PAL and UFGT activity assays, the protein extraction was performed according to the methods of Mori et al. (2005, 2007), respectively. Peroxidase activity was instead measured on berry skin after protein extractions as described by Ushimaru et al. (1997).

For PAL activity measurement, the reaction mixture consisted of 0.5 ml of phenylalanine and 0.5 ml of protein extract. The assay mixture was incubated at 37°C for 60 min. The reaction was terminated by adding 0.5 ml of HCl acid (18%). The quantity of the product, *trans*-cinnamic acid, was calculated using its extinction coefficient of 9630 M^-1^cm^-1^ at 290 nm. One unit (U) of PAL activity, expressed on berry skin fresh weight, was defined as the production of 1 mol of *trans*-cinnamic acid per minute.

The UFGT assay was performed on the protein extract using either cyanidin or delphinidin as substrate in 200 mM Tris-HCl (pH 7.5), containing 0.1 mM cyanidin or delphinidin and 10 mM UDP-glucose. After incubation for 5 min at 37°C, the reaction was stopped by adding 150 μl 5% HCl. The concentration of cyanidin-3-glucoside and delphinidin-3-glucoside was calculated at 520 nm and pH 1, using extinction coefficients of 26,900 M^-1^cm^-1^ and 26,000 M^-1^cm^-1^, respectively. One unit (U) of UFGT activity, expressed on berry skin fresh weight, was defined as the production of 1 mol of cyanidin-3-glucoside or delphinidin-3-glucoside per second.

Guaiacol peroxidase activity was determined in the ripening berry skin as described by Ushimaru et al. (1997), using pyrogallol as the electron donor. The reaction mixture comprised the protein extract in 50 mM sodium phosphate buffer (pH 7.0), 0.1 mM H_2_O_2_ and 50 mM pyrogallol (H_2_O_2_ and pyrogallol were freshly prepared just before use). The absorbance at 430 nm was recorded immediately after the addition of pyrogallol and after 7 min at room temperature, and was compared to a blank with no protein extract added. One unit (U) of peroxidase activity, expressed on berry skin fresh weight, was defined as the amount of enzyme that catalyzes the oxidation of 1 μmol of pyrogallol per minute.

### Statistical Analyses

The experiment had a completely randomized design and the agronomic parameters and biochemical data were submitted to analysis of variance (ANOVA) using SAS statistical software (SAS Institute, Cary, NC, United States) with four replications for each treatment. The temperature data were analyzed with 10 replications for each treatment.

### RNA Extraction and Microarray Analyses

Total RNA was isolated from approximately 400 mg of pulverized berry skins from three biological replicates sampled at all dates except harvest, for a total of 27 berry samples [3 control samples + (2 thermal regimes × 4 stages × 3 biological replicates)], using the Spectrum Plant Total RNA kit (Sigma–Aldrich), with modifications as described in Dal Santo et al. (2016). RNA quality and quantity were determined using a Nanodrop 2000 spectrophotometer (Thermo Scientific, Wilmington, DE, United States) and a Bioanalyzer Chip RNA 7500 series II (Agilent, Santa Clara, CA, United States). We hybridized 5 μg of total RNA per sample to a NimbleGen microarray 090818_Vitus_exp_HX12 chip (Roche, NimbleGen Inc., Madison, WI, United States), according to the manufacturer’s instructions (Dal Santo et al., 2013). Statistical analysis of the microarray data was conducted using TMeV v4.8^1^. Statistical analysis of microarrays (SAM) was performed with a false discovery rate (FDR) of 0.1% and ANOVA using α = 0.05 and standard Bonferroni correction. Heat maps were created using log2-transformed expression values and then median-centered by transcript. Cluster analysis was performed by the k-means method (KMC) with Pearson’s correlation distance. Principal component analysis (PCA) was conducted using SIMCA P+ v13 (Umetrics, United States). Gene Ontology (GO) annotation was applied using the BiNGO v2.3 plug-in tool in Cytoscape v2.6 with PlantGOslim categories, as described by Maere et al. (2005). Overrepresented PlantGOslim categories were identified using a hypergeometric test with a significance threshold of 0.05. STEM v1.3.8 was used for clustering, comparing and visualizing gene expression data (Ernst et al., 2005).

### Reverse Transcription (RT) and Real Time qPCR

One microgram of extracted RNA was treated with 2 units (U) of Turbo DNase (TURBO DNA-free kit—Ambion) according to the instructions provided with the commercial kit. DNase- treated RNA was then used for cDNA synthesis using the SuperScriptIII Reverse Transcriptase kit (Invitrogen) following the producer’s indications. In order to assess if the cDNA had been properly produced, an amplification with primers designed on the 3′UTR of an actin coding gene (VIT_12s0178g0020, (Pastore et al., 2011) was performed. Real Time qPCR was performed using GoTaq^®^ GreenMaster Mix kit (Promega) to amplify a specific region of target genes (UFGT, VvUFGT – VIT_16s0039g02230; PAL – VIT_00s2849g00010 and the peroxidase VvPrx31 – VIT_14s0066g01850) with previously described primer pairs (Movahed et al., 2016). Primers and cDNA were mixed with the Power SYBR^®^ Green PCR Master Mix (Applied Biosystems, Foster City, CA, United States) and the reaction was carried out on an ABI PRISM StepOne Sequence Detection System (Applied Biosystems, Foster City, CA, United States) using the following cycling conditions: 95°C hold for 10 min followed by 45 cycles at 95°C for 30 s, 55°C for 30 s and 72°C for 20 s. 95°C hold for 2 min followed by 40 cycles at 95°C for 15 s, 55°C for 30 s, 60°C for 30 s, and 95°C for 15 s. Non-specific PCR products were identified by the dissociation curves. Amplification efficiency was calculated from raw data using LingReg PCR software (Ramakers et al., 2003). The mean normalized expression (MNE)-value was calculated for each sample referred to the ubiquitin expression according to the Simon equation (Simon, 2003). Standard error (*SE*) values were calculated according to Pfaffl et al. (2002).

### Accession Numbers

Grape berry microarray expression data are available in the Gene Expression Omnibus under the series entry GSE92864^2^.

## Results

### Two Different Thermal Regimes Differently Affect Ripening Parameters

**FIGURE 1:**
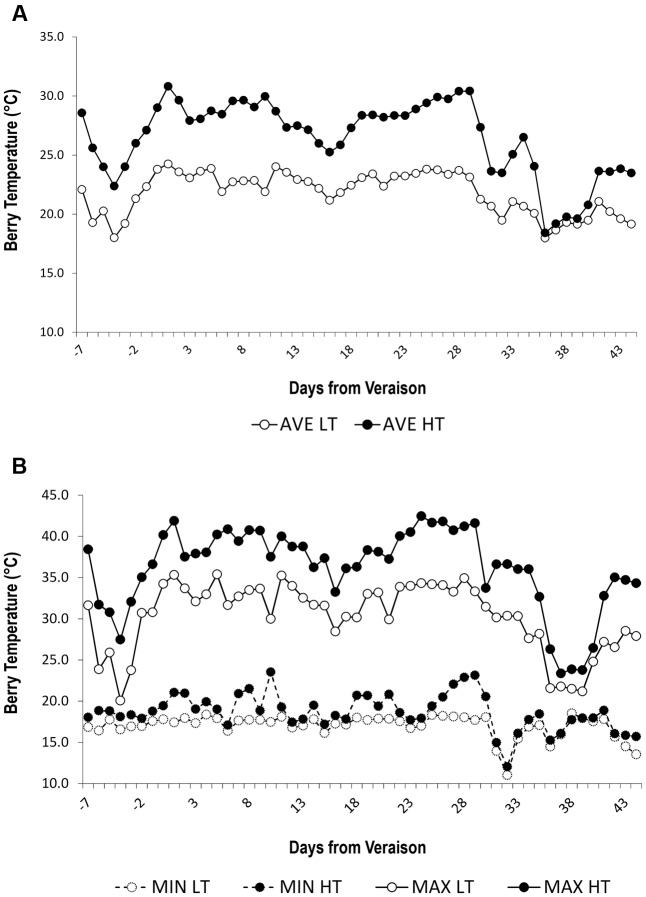
Daily berry temperature trends. **(A)** Average and **(B)** minimum (dashed line) and maximum (whole line) daily berry temperature (°C) trends under two temperature regimes. LT = low temperature = white symbol, HT = high temperature = black symbol.

We set up an experimental design to impose two different thermal regimes in potted grapevine plants over the course of grape ripening. Five vines were placed in a plastic greenhouse where the air temperature was artificially cooled whereas other five vines were placed in an identical plastic greenhouse with only the basal segment open. These two conditions were named LT and HT, respectively, with HT representing the closest condition to the 2012 thermal regime. HT and LT berry temperature were strongly differentiated in the two greenhouses (**Table 2** and **Figure 1**). Indeed, the average berry temperature during the treatment period was ∼21.8°C in the LT greenhouse and 26.5°C in the HT greenhouse (**Table 2** and **Figure 1A**). Maximum temperatures were higher in HT during ripening, with several heatwaves, sometimes reaching values of around 40°C in HT berries, corresponding to 7–8°C higher than the maximum temperatures detected in LT berries (**Figure 1B**). Overall, HT berries accumulated an additional 238 Degree Days (DD) compared to LT berries (**Table 2**). The number of hours with berry temperature exceeding 30°C was four-fold greater in the HT greenhouse, while berry temperature exceeding 35°C was registered only in the HT greenhouse (**Table 2** and **Figure 1B**). On the contrary, the minimum temperatures in HT and LT were aligned (**Figure 1B**).

**Table 2:** Average Berry Temperature, accumulated Degree Days (DDs), and heatwaves hitting the berries (calculated as the number of hours with average berry temperature > 30°C and > 35°C) under the two different growing regimes.

Treatment	Average berry temperature (°C)	Accumulated DDs	Average berry temperature > 30°C	Average berry temperature > 35°C
HT	26.5	850	392	157
LT	21.8	612	91	0
	^∗^	^∗^	^∗^	^∗^


*Values represent means of ten replicates. LT = low temperature, HT = high temperature. Asterisks indicate significant differences between HT and LT using ANOVA (^∗^*P* < 0.05).*

**FIGURE 2:**
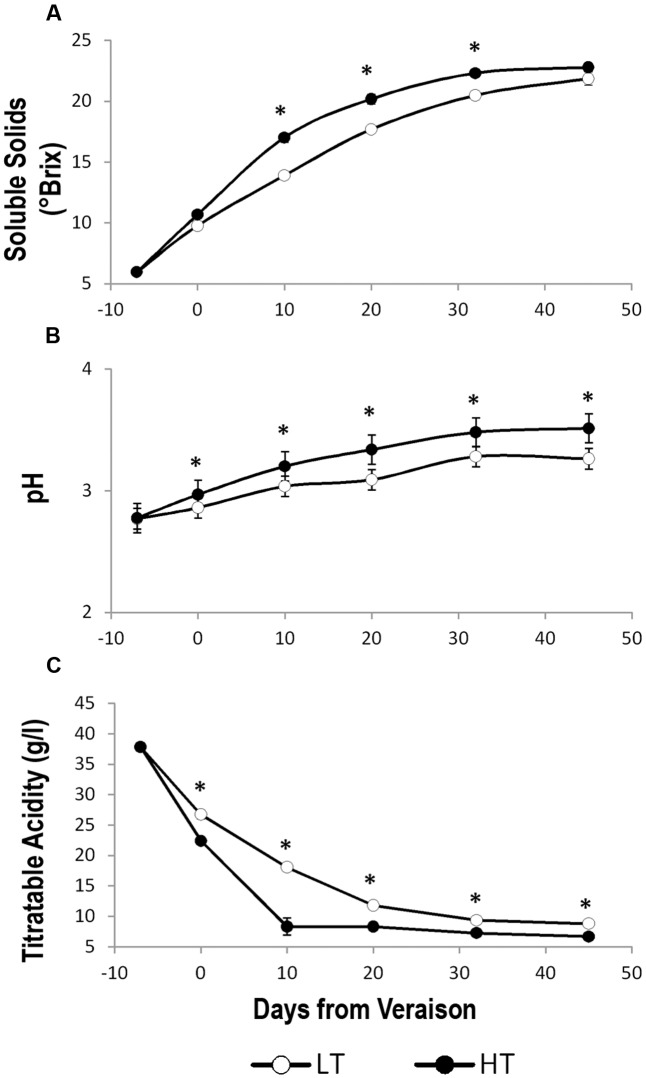
Trends of the main berry ripening parameters under different temperature regimes. Trends of **(A)** soluble solids (°Brix), **(B)** pH, and **(C)** titratable acidity in grape berry samples grown under two different temperature regimes. LT = low temperature = white symbol, HT = high temperature = black symbol. Bars represent ± SE (*n* = 4). Asterisks indicate significant differences between HT and LT at the same date using ANOVA (^∗^*P* < 0.05).

The LT thermal regime slightly delayed technological ripening in berries, which showed a lower level of °Brix and pH and higher values of titratable acidity compared to HT. However, at harvest, the soluble solids reached comparable values in HT and LT berries (**Figure 2**).

**FIGURE 3:**
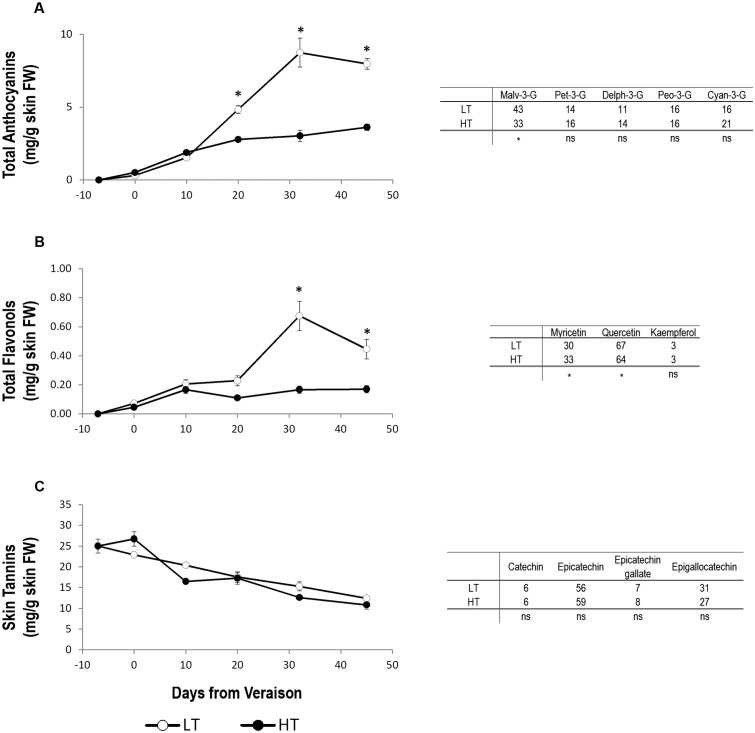
Concentration trends and relative composition of the main flavonoid compounds at harvest. Total concentration trends (left panels) and relative composition at harvest (right tables) of anthocyanins **(A)**, flavonols **(B)**, and skin tannins **(C)** in grape berry samples grown under two different temperature regimes. LT = low temperature = white symbol, HT = high temperature = black symbol. Bars represent ± SE (*n* = 4). Asterisks indicate significant differences between HT and LT at the same date using ANOVA (^∗^*P* < 0.05). ns, not significant.

Until 10 days after veraison, there was no detectable difference in total anthocyanin concentration between the two thermal regimes (**Figure 3A**). Starting from 10 days after veraison, total anthocyanin accumulation rate began to accelerate in the skin of LT berries compared to HT. This different behavior was maintained at harvest, when the concentration of total anthocyanins in the skin of LT grapes was almost doubled compared to HT (**Figure 3A**). The most abundant individual anthocyanin in Sangiovese berry skin was malvidin-3-glucoside, in both LT and HT treatments (**Figure 3A**). However, its percentage was significantly lower in HT than LT (**Figure 3A**). This decrease of malvidin-3-glucoside was counterbalanced in HT by an increase in the percentage of petunidin-3-glucoside, delfinidin-3-glucoside, and especially of cyanidin-3-glucoside, albeit non-significant.

The accumulation trend of flavonols was similar to that of anthocyanins in both HT and LT. At harvest, flavonol concentration in LT berry skin was thus three-times of that found in HT berry skin (**Figure 3B**). The analyses of the single flavonol compounds at harvest showed a higher quercetin and lower myricetin percentage in LT berries compared to HT (**Figure 3B**). Kaempferol was instead unaffected by different growing temperature regimes (**Figure 3B**).

Berry skin tannins decreased during ripening without significant differences between HT and LT in any developmental stage analyzed. Likewise, regardless of thermal regime, the percentage, measured at harvest, of the four individual flavan-3-ol monomers catechin, epicatechin, epicatechin-gallate and epigallocatechin did not differ between HT and LT berries, epicatechin being the tannin compound present in the greatest concentration in both treatments (**Figure 3C**).

Our results suggest a different effect of LT and HT thermal regimes, to a less extent, on technological berry ripening and, more strongly, on flavonoid increase, with the enhancement of anthocyanin and flavonol accumulations under LT conditions.

### The Effect of Temperature on Berry Skins Whole Transcriptome

The transcriptome of Sangiovese berry skins under LT and HT growing temperature regimes was assessed at five sampling times (i.e., before the treatments (T0), at veraison (T1) and 10 (T2), 20 (T3) and 32 (T4) days after veraison). The dataset was initially screened by Significance Analysis of Microarrays (SAM, 9 groups, FDR = 0.1%) to select genes that were differentially modulated under our experimental conditions. Analysis of Variance (ANOVA, 9 groups, α = 0.05, standard Bonferroni correction) was applied to transcripts positive in the SAM in order to retrieve the most significantly modulated transcripts (6441 genes, Supplementary File 1).

**FIGURE 4:**
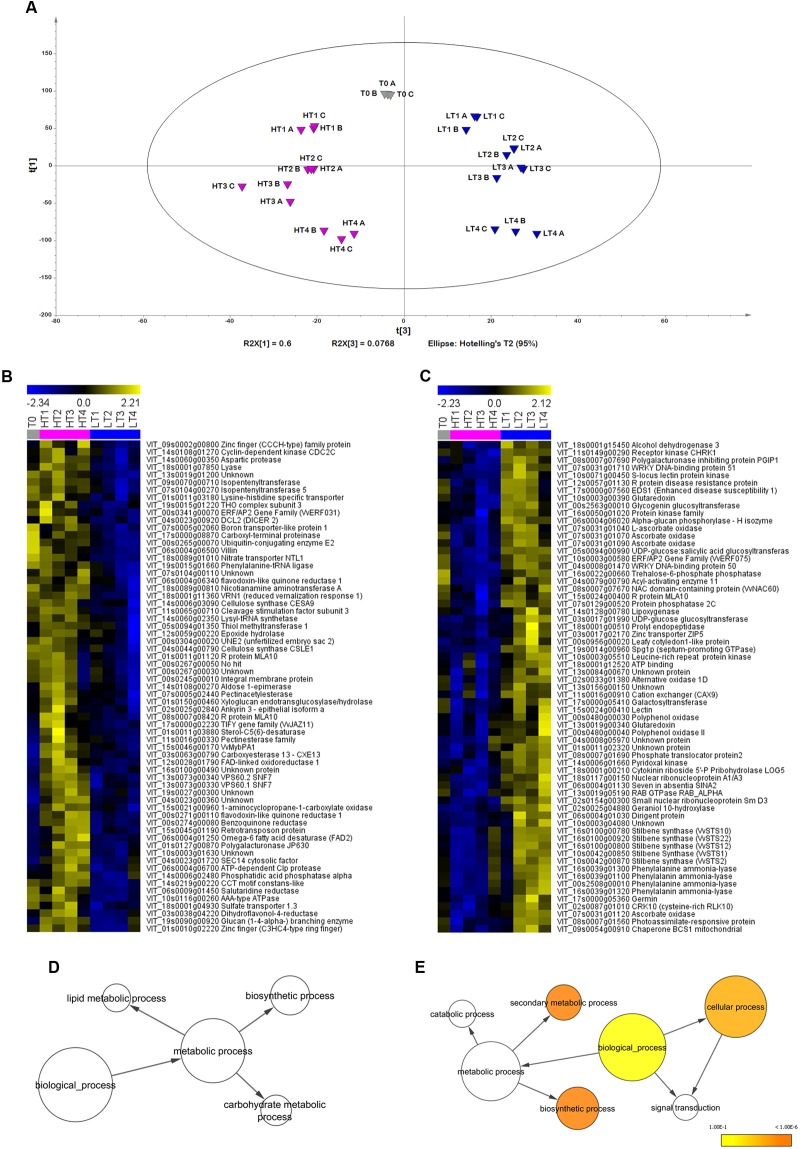
Global gene expression trends in Sangiovese berries cultivated under two different temperature regimes. **(A)** Score scatterplot (PC3 vs. PC1) of the PCA model [8 Principal Components, *R*^2^(cumulative) = 0.941, *Q*^2^(cumulative) = 0.848] applied to the significantly modulated transcripts dataset. **(B,C)** Expression profiles of genes positively (right) and negatively (left) correlated to the third principal component were selected within the first (positive) and last (negative) percentile of the third component loadings. Enriched GO terms for the genes negatively **(D)** and positively **(E)** correlated to the PC3. The network graphs show BiNGO visualizations of the overrepresented GO terms. Categories in GoSlimPlants (Maere et al., 2005) were used to simplify this analysis. Colored nodes represent GO terms that are significantly overrepresented (*p* < 0.05). T0 = plants before treatment, LT = low temperature, HT = high temperature. Sample names are composed by temperature treatment abbreviation followed by the indication of the developmental stage (1, 2, 3, or 4), and by the description of the biological replicate **(A–C)**. Gray, blue, and purple indicate samples of control, LT-treated and HT-treated, respectively.

To verify the uniformity of biological replicates and investigate the transcriptomes of LT and HT berries, we performed a PCA, obtaining a significant model (8 PCs, R2X = 0.941, Q2 (cum) = 0.848, **Figure 4A**). PC1 explained ∼60% of the total dataset variability and mostly reflected differences among the four sampling dates (**Figure 4A** and Supplementary Figure 2), suggesting a slight delay in LT berry ripening at stages 3 and 4 in comparison to berries ripened under HT conditions. PC2 accounted for 13.3% of the total variability and mainly described differences between first and last sampling times, and intermediate ones (Supplementary Figure 2). Notably, PC3, explaining ∼8% of total dataset variability reflected differences among T0, LT and HT samples (**Figure 4A**). Indeed, the expression profiles of the first and last percentile PC3 loadings showed more expression in LT and HT samples, respectively (**Figures 4B,C**). The GO enrichment analysis revealed that the positive PC3 loadings were significantly enriched in the functional categories Cellular process, Biosynthetic process and Secondary metabolic process (**Figure 4E**). In particular, the first percentile PC3 loadings, more expressed in LT, comprised several genes involved in the metabolism of phenylpropanoids (5 Stilbene Synthase, STS, and 4 PAL), in the plant response to abiotic and biotic stresses (several ascorbate oxidases and glutaredoxines as well as many R proteins), in carbohydrate metabolism (one alcohol dehydrogenase and one trehalose-6-phosphate phosphatase). The transcript of ethylene response factor VvERF075, which belongs to the AP2/ERF superfamily and is usually upregulated in berry skin during ripening (Licausi et al., 2010), was also found more abundant in LT. Furthermore, we found among the positive PC loadings the transcription factor VvNAC60 already described as a putative master regulator of the transition between unripe and ripe red berries (Palumbo et al., 2014).

No significant GO enrichment could be found in the negative loadings of the PC3, more expressed under HT regime (**Figure 4D**). The last percentile of the PC3 loadings showed higher level of transcript under HT regime of several cell-wall related transcripts (including two cellulose synthases, a pectinacetylesterase and a xyloglucan endotransglucosylase/hydrolase), of regulatory and structural genes involved in cytokinin metabolism (two isopentenyltransferases) and in ethylene metabolism (the 1-aminocyclopropane-1-carboxylate oxidase), in protein degradation and biosynthesis (including several proteases and amino acid transporters) and in the metabolism of carbohydrates and lipids (including an aldose 1-epimerase and a desaturase). Two genes involved in flavonoid biosynthesis and regulation, the dihydroflavonol-4-reductase and the transcription factor VvMybPA1, previously reported as a regulator of proanthocyanidin biosynthesis (Bogs et al., 2007) were also more expressed in HT compared to LT.

In summary, the comparison of berry skin transcriptomes highlighted the effect of the temperature on gene expression during fruit ripening. In particular, LT condition was characterized by a higher expression of transcripts associated with the metabolism of phenylpropanoids.

### Gene Expression Profiles Inspection Highlights a Strong Effect of Temperature on Early Phenylpropanoid Pathway

**FIGURE 5:**
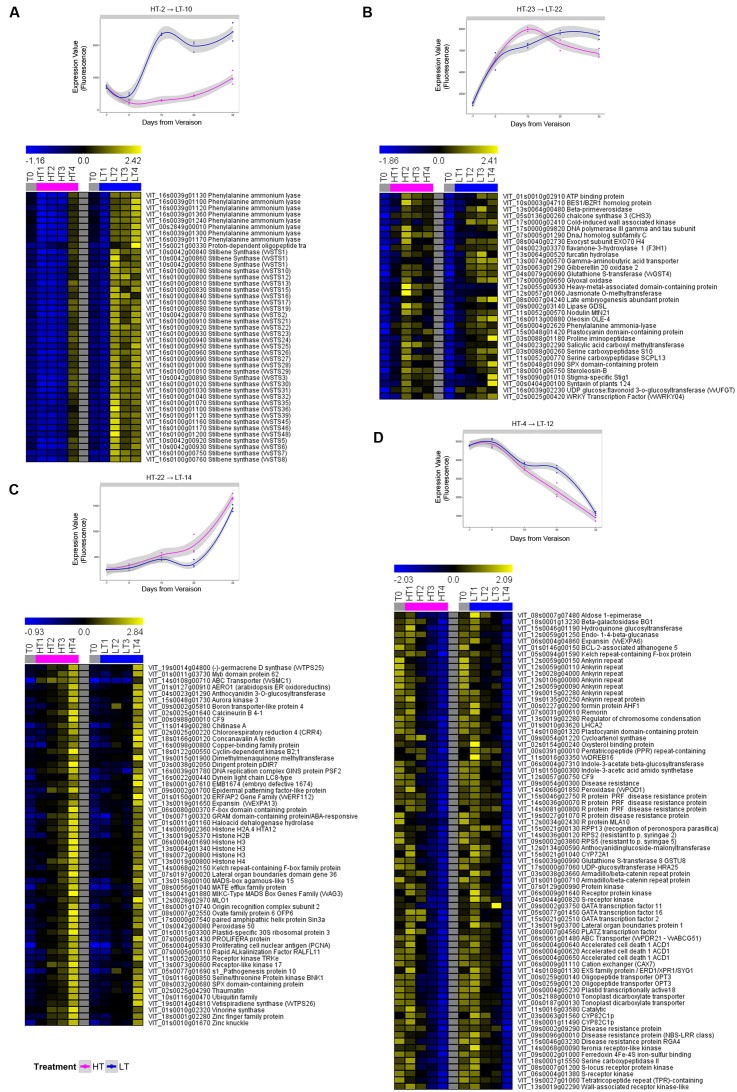
Growing temperature regime affects specific clusters of genes. Four selected significant profiles (<5% Bonferroni correction method) of the 2,257 genes modulated in different growing temperature, from among 25 profiles obtained by STEM analysis. For each cluster, the average gene expression trend (top panels) and heat map of all the genes’ expression profiles (bottom panels) are depicted. See Supplementary File 3 for the complete comparison profile table and clusters numbering. **(A)** HT 2 → LT 10, **(B)** HT 23 → LT 22, **(C)** HT 22 → LT 14, **(D)** HT 4 → LT 12. T0 = plants before treatment, LT = low temperature, HT = high temperature. Sample names are composed by temperature treatment abbreviation followed by the indication of the developmental stage (1, 2, 3 or 4). Data are the average of the three biological replicates. Gray, blue, and purple indicate samples of control, LT-treated and HT-treated, respectively.

We focused on changes in expression profiles of genes scoring an absolute value of fold change |FC|≥ 2, identifying 2,257 annotated genes (Supplementary File 2). Differences in the timing of activation/repression of the differentially expressed genes during ripening in HT and LT was investigated in more detail by applying the short time-series expression miner (STEM) clustering method (Ernst et al., 2005) to the 2,257 identified genes (Supplementary File 3). **Figure 5** reports the most significant results of the STEM approach. Some of the transcriptional trends changes were also confirmed by Real Time qPCR analysis (Supplementary Figure 3).

Notably, many PAL and STS genes resulted as much more expressed in LT berry skins after veraison (**Figure 5A**), whereas they showed a slower rate of activation in HT berry skins (HT-2 → LT-10 in STEM analysis – Supplementary File 3), corroborating the PCA results (**Figure 4A**). Interestingly, a similar trend of expression was also detected for a proton-dependent oligopeptide transporter (POT), which contains a PTR2 domain that characterizes both nitrate and peptide transporters (Widhalm et al., 2015).

We found transcripts showing a peak of expression 10 days after veraison in HT, while their expression generally increased during the whole ripening period in LT (**Figure 5B**; HT-23 → LT-22 in STEM analysis – Supplementary File 3). Some genes involved in anthocyanin biosynthesis and transport (VvUFGT and VvGST4) and other phenylpropanoid/flavonoid related genes (one PAL, VvCHS3 and VvF3H1), were found to belong to this group, together with transcripts involved in hormone metabolism, such as one jasmonate O-methyltransferase and one gibberellin 20 oxidase, and with one gene that showed high homology with a cold-induced wall associated kinase (Cao et al., 2009).

We instead found that in HT berry skins, starting from 10 days after veraison, there was an earlier activation (HT-22 → LT-14 in STEM analysis – Supplementary File 3) of genes involved in the biosynthesis of volatile aromas [the terpene synthases VvTPS25 and VvTPS26, (Martin et al., 2010)], in cell wall metabolism and in DNA metabolism (**Figure 5C**). The same trend was shared by one peroxidase transcript (peroxidase 50).

Several genes that were downregulated in both HT and LT berries during ripening showed a different expression profile, i.e., a rapid and progressive decrease of expression starting from veraison in HT berries, and a much slower decrease in LT berries (**Figure 5D**; HT-4 → LT-12 in STEM analysis – Supplementary File 3). Cell wall-related transcripts, including genes linked to chlorophyll degradation and response to biotic stresses showed this trend. *VvPrx31*, a gene coding for a peroxidase putatively associated with anthocyanin degradation (Movahed et al., 2016) was also found in this group.

**FIGURE 6:**
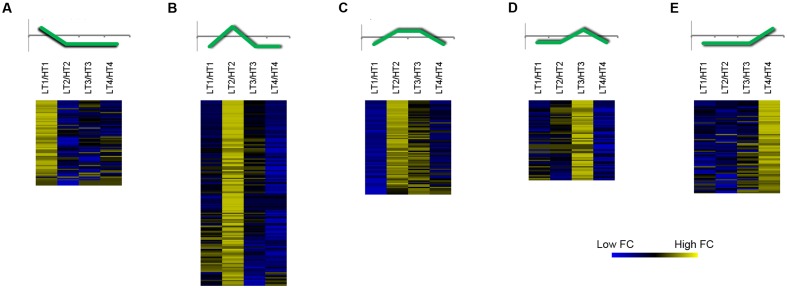
Clustering analysis of the most differently expressed genes between Low (LT) and High (HT) growing temperature regime. Heat maps of the Fold Change (FC) of highly expressed (threshold value of raw fluorescence intensity ≥ 100) and modulated genes (|FC| ≥ 2) between LT and HT in at least one stage of development. Five clusters **(A–E)** were obtained by applying a K-Means Clustering analysis (KMC, Pearson’s correlation distance). A schematic representation of the general FC trend is depicted on top of each cluster heat map. Sample names are composed by temperature treatment abbreviation followed by the indication of the developmental stage (1, 2, 3 or 4). Data are the average of the three biological replicates.

In order to point out the genes differentially expressed between LT and HT, we decided to consider just genes with a fluorescence expression threshold value ≥ 100 and transcripts in which the |FC| between LT and HT was ≥ 2 in at least one stage of development. By this approach, we obtained 417 differentially expressed genes (Supplementary File 4) that were over-represented in the GO functional categories of Secondary metabolic process, Generation of precursor metabolites and energy, Response to biotic stimulus, and Biosynthetic process (Supplementary Figure 4). These genes were grouped into five different FC clusters by K-means clustering (KMC) analysis highlighting the times with the greatest difference in gene expression between LT and HT (**Figure 6**). At a glance, the genes in each cluster showed a peak of expression at a given stage in LT. In the other ripening stages, expression of the same genes may be higher in HT, however, the FC of the LT/HT ratio at the peaking stage was generally much higher compared to the HT/LT values in the other stages. The most represented cluster included genes with high FC between LT and HT at 10 days after veraison (**Figure 6B**). The remaining differentially expressed genes were almost equally divided in the other four clusters (**Figure 6**). In the first cluster (**Figure 6A**), collecting genes more promptly activated by the LT regime at veraison (stage 1), we found a glycerol-3-phosphate acyltransferase 3 (AtGPAT3) to be the most differentially expressed gene (Supplementary File 4). Furthermore, several ERF/AP2 transcription factors (Licausi et al., 2010) were among the most differentially expressed genes belonging to this cluster (**Figure 6A** and Supplementary File 4). STSs were the most differentially expressed genes in LT regime at 10 and 20 days after veraison (**Figures 6B,C**), confirming the strong involvement of such genes in association with LT regime, as previously observed with the STEM approach (**Figures 4C**, **5A**). In these clusters, we also found genes involved in the response to biotic stimuli, such as wound induced proteins, beta 1–3 glucanase, and pathogenesis-related proteins, indicating a general activation of defense mechanisms under LT conditions. The cluster in **Figure 6D** is characterized by high FC in LT/HT 20 days after veraison (Supplementary File 4). Interestingly, this cluster included three ABC transporters and three isoforms of LRR receptor kinase CLAVATA1 (CLV1), which seem to be able to confer resistance to various abiotic stresses (Grzeskowiak et al., 2013). Lastly, several genes involved in volatile compounds synthesis were present in the last cluster, which groups genes showing a peak of expression in LT at 32 days after veraison (**Figure 6E** and Supplementary File 4). Two galactinol synthases, were also included in this cluster.

Overall, using this FC clustering approach we were able to validate expression of genes related to phenylpropanoid biosynthesis identified by STEM analysis, but also to retrieve other genes involved in the metabolism of volatile compounds, lipids and hormones that showed high sensitivity to thermal changes.

### Temperature Affects the Activity of Enzymes Involved in Anthocyanin Metabolism

**FIGURE 7:**
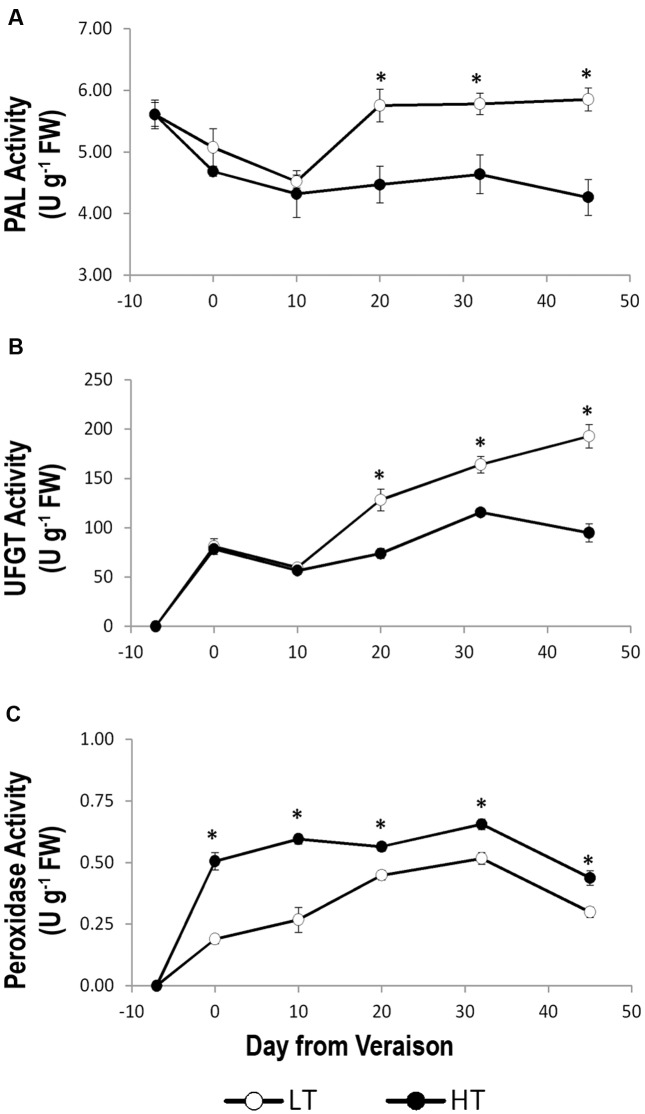
Activity of enzymes involved in anthocyanins biosynthesis, phenylalanine ammonia-lyase (PAL, **A**) and UDP glucose:flavonoid 3-o-glucosyltransferase (UFGT, **B**), and putatively involved in anthocyanins degradation (peroxidases, **C**) in grape berry samples grown under two different temperature regimes. LT = low temperature = white symbol, HT = high temperature = black symbol. Bars represent ± SE (*n* = 4). Asterisks indicate significant differences between HT and LT at the same date using ANOVA (^∗^*P* < 0.05).

In LT treatment, growing temperatures positively impacted on the anthocyanins accumulation compared to HT (**Figure 3A**). However, such an accumulation was not fully explained by differences in transcription of genes related to phenylpropanoid/flavonoid metabolism. We therefore evaluated the enzymatic activity of PAL, the key enzyme of phenylpropanoid biosynthesis pathway (Zhang and Liu, 2015), of UFGT, which catalyzes the last step of anthocyanin biosynthesis and is considered the key enzyme for this pathway (Kobayashi et al., 2001), and of peroxidases, which are supposed to degrade anthocyanins under elevated temperature growing conditions (Mori et al., 2007; Movahed et al., 2016). PAL activity gradually decreased in both temperature regimes until 10 days after veraison and was steady thereafter in HT, whereas it abruptly increased in LT, especially between 10 and 20 days after veraison. Afterward, high levels of PAL activity still persisted in LT berries. Thus, from the end of veraison to harvest, PAL activity under HT conditions was significantly lower in berry skins in comparison to LT (**Figure 7A**).

In order to elucidate a possible effect of the two thermal regimes on the affinity of UFGT for delphinidin or cyanidin, the enzymatic assay was performed twice using both substrates (**Figure 7B** and Supplementary Figure 5). After an initial fluctuating trend, in both cases the activity of UFGT showed an increasing trend from 10 days after veraison to harvest in HT and LT berries (**Figure 7B** and Supplementary Figure 5) and a strong and significant reduction of UFGT activity was observed in HT compared to LT from 20 days after veraison to harvest. No differences were found in terms of higher or lower affinity of the UFGT enzyme for delphinidin or cyanidin substrates as the activity of the enzyme showed comparable values between the two assays after veraison (**Figure 7B** and Supplementary Figure 5). The peroxidase activity of HT and LT berry skin showed a similar trend during ripening, with a significantly higher activity in HT than LT berries from veraison to harvest (**Figure 7C**), supporting a role of peroxidases in anthocyanins degradation under HT conditions.

Overall, the enzymatic activity analyses indicated the presence of a different balance between anthocyanin biosynthesis and degradation under LT and HT thermal regimes due to an increased action of PAL and UFGT enzymes in LT and of peroxidases in HT.

## Discussion

### Increased Temperature Accelerates Sugar Accumulation and Acidity Depletion during Ripening

The increasing temperature associated with climate change is expected to modify air and land temperatures in most vine growing regions, which will undergo a warming of 2 to 4°C in the next decades (Hannah et al., 2013). Mild to moderate temperature increases were shown to cause phenological changes in grapevine, accelerating the vegetative development and fruit maturation, ultimately affecting the berry composition (Duchene and Schneider, 2005; Jones et al., 2005). The two regimes analyzed in this study (LT and HT) can be considered representative of the inter-seasonal thermal variability occurring in recent years in the typical Sangiovese growing area of north-central Italy (Filippetti et al., 2013; Teslić et al., 2016), with a tendency toward the thermal level of HT. The experiment design aimed to affect temperature regimes, without modifying other environmental parameters. The main differences between HT and LT regimes during pre-veraison-to-harvest period were mainly associated to an increase in maximum temperature under HT conditions. There was a general acceleration of sugar accumulation and total acidity reduction in the HT thermal regime. The effect of elevated temperature on sugar accumulation may depend on the amount of temperature variation as it has been reported that HTs (≥40°C) could impact on the photosynthetic supply of sugar to the berry, causing a significant reduction in sugar accumulation (Greer and Weston, 2010; Greer and Weedon, 2013). On the contrary, the milder conditions of our experiment, in which temperatures ≥ 40°C were seldom recorded under HT regime, led to an increased sugars accumulation, compared to LT, in all developmental stages except at harvest time. Indeed, a decrease in acidity and increase in pH associated with HT have been reported in other grape varieties (i.e., Shiraz, Chardonnay and Cabernet Franc) grown under warm conditions (Sadras and Moran, 2012; Bonada and Sadras, 2015). However, other studies registered no effect of temperature increase on either titratable acidity or pH, nor on both simultaneously (Sadras and Moran, 2012; Greer and Weedon, 2013; Movahed et al., 2016) corroborating a previous hypothesis that grape berry acidity and pH depend on the interaction between cultivar and the amount of temperature increase (Sadras and Moran, 2012).

### Increased Temperature Negatively Affects Anthocyanin and Flavonol Concentrations

The influence of temperature on the flavonoid concentration of grape berry has been extensively reviewed (Downey et al., 2006; Teixeira et al., 2013). The biosynthesis of tannin and flavonols is high at flowering and in the berry skin the accumulation increases from fruit set until 1 to 2 weeks after veraison (Kennedy and Jones, 2001; Downey et al., 2003; Bogs et al., 2005). Anthocyanin accumulation, on the contrary, starts from veraison and reaches its maximum in the latest phases of fruit maturation, when their synthesis ceases (Boss and Davies, 2001). In our study, no significant relationship could be verified between temperature increase and total skin tannin concentration in Sangiovese berries (**Figure 3**). These results, in line with the findings by Cohen et al. (2012), suggested that increasing temperature from veraison to harvest has little impact on tannin accumulation, probably because these compounds have already been synthesized. Furthermore, skin tannins are the most stable flavonoids under diverse growing conditions, due to their chemical structure which is widely variable in size, ranging from dimers to polymers with more than 40 units (Teixeira et al., 2013). This could cause less susceptibility to potential degradative processes induced by temperature.

The HT thermal regime induced a similar decrease in the concentration of flavonols and anthocyanins (**Figure 3**). Flavonols are very sensitive to changes in environmental conditions. For example, sunlight is known to enhance flavonols accumulation in berries (Downey et al., 2006), reflecting their role as UV protectants (Spayd et al., 2002; Pastore et al., 2013). Since we maintained the same light intensity and quality between the two greenhouses, our data highlighted the strong temperature effect on this class of compounds. The reduced accumulation of anthocyanins in Sangiovese berries during ripening under increased temperature has been already reported for various genotypes in different conditions (Spayd et al., 2002; Mori et al., 2005, 2007; Tarara et al., 2008; Movahed et al., 2016).

Anthocyanins and flavonols changed also their composition, in berry skins. In particular, HT berries had a lower percentage of the anthocyanin malvidin 3-G and the flavonol quercetin. In grapevine, growing temperatures have been associated with increased proportions of highly hydroxylated and methylated anthocyanins (Mori et al., 2007; Tarara et al., 2008; Cohen et al., 2012). Conversely, in a recent study an increase in the degradation of petunidin and malvidin glucoside at elevated temperatures was observed in Cabernet Sauvignon grapes exposed to labeled phenylalanine (Chassy et al., 2015). The relationship between berry temperature and flavonols profiles has been less extensively studied. Consistently with our results, a higher proportion of flavonols with di-hydroxylation, as quercetin, was detected in Merlot berries when temperature was reduced by approximately 8°C in comparison with control temperature (Cohen et al., 2008).

Overall, this evidence supports the high susceptibility of anthocyanins and flavonols to air temperature, and the critical role of experimental conditions in this kind of assessment. Accumulation of these classes of secondary compounds is the result of complex and interconnected processes such as synthesis, degradation, hydroxylation, methylation, acylation and transport, thus it is not always possible to determine general univocal relations.

### Transcriptomic Analysis Highlights Processes Affected by Temperature in Berry Skin

We analyzed the entire transcriptome of the berry skin in cv. Sangiovese during ripening exploiting a combination of complementary statistical approaches to retrieve those transcripts mostly associated with each of the two temperature regimes. In particular, the PCA analysis suggested that, beyond a slight transcriptional hastening of ripening in a few developmental stages of HT samples, a clear rearrangement in the skin transcriptome (∼8% total variability of the dataset) can be ascribed to the imposition of different thermal regimes, throughout the course of the experiment.

The inhibitory effects of HT on stilbene biosynthetic pathway have already been described by Rienth et al. (2014) and in our conditions many members of the STS and PAL gene families were induced under LT regime suggesting a clear activation of stilbene biosynthesis. A coordinated gene expression of PAL and STS was observed in grape berry, suggesting that several enzymatic steps in the stilbene biosynthetic pathway are co-regulated (Zenoni et al., 2016).

Interestingly, several ERF transcription factors resulted as promptly higher expressed under LT compared to HT regime, whereas few were less expressed under LT regime, suggesting that members of this family of transcription factors, known regulators of thermotolerance in plants (Carbonell-Bejerano et al., 2013) play a central role in the response to temperature variation in grapevine berry skin as reported for Arabidopsis (Cheng et al., 2013), Chickpea (*Cicer arietinum*) (Deokar et al., 2015) and tomato (Severo et al., 2015). Moreover, the presence of ACO1 and ACS among genes more expressed in LT suggests that the ethylene signaling is required in the regulation of the temperature-driven ripening processes of grapevine berries. Another response observed under LT regime was associated to the reaction toward environmental solicitations. This included the glycerol-3-phosphate acyltransferases (GPAT) that catalyze the acylation at sn-1 position of glycerol-3-phosphate to produce lysophosphatidic acid (LPA), an important intermediate for the formation of different types of acyl-lipids (Chen et al., 2011) and two galactinol synthases, whose involvement in multiple abiotic stresses responses (Zhuo et al., 2013) and in particular in heat stress responses (Pillet et al., 2012) has been reported.

Lastly, some genes involved in the biosynthesis of aromas, such as linalool synthase, delta-cadinene synthase, vetispiradiene synthase and a germacrene synthase, were found more expressed under LT conditions. These results well corroborate previous reports regarding the reduction in aromatic potential in grapevine berries exposed to HT (Rienth et al., 2014).

### Transcriptional and Post-transcriptional Regulation of Genes Belonging to the Phenylpropanoid/Flavonoid Pathway

The accumulation of flavonoids and anthocyanins in Sangiovese berries skin was strongly affected by the two thermal regimes. However, a clear correlation between the upregulation of genes involved in anthocyanin and flavonol biosynthesis and transport and the higher levels of these compounds under LT conditions was not revealed. The slight difference of the VvUFGT and VvGST4 expression profile in LT compared to HT seems insufficient to support the significant difference in anthocyanin accumulation. Similar results were obtained by other authors reporting that mRNA accumulation of anthocyanin biosynthetic genes was not reduced under HT growing conditions (Mori et al., 2007; Carbonell-Bejerano et al., 2013; Rienth et al., 2014). Moreover, the expression of VvMYBA1, the transcription factor that activates VvUFGT and VvGST4 in grape berry skin, was not significantly affected by the thermal regime, under our experimental conditions. A first hypothesis to explain the strong differences observed in terms of flavonoid accumulation between LT and HT, based on the expression profiles of other genes involved in such metabolism, suggests the involvement of the proanthocyanidin regulator VvMybPA1 in the activation of an alternative branch under HT regime, competing with anthocyanins pathway. Another hypothesis arose from the high expression of several PAL members in LT conditions, which could supply substrates to both stilbenes and, presumably, flavonoid accumulation. An additional possibility derives from the observation that the enzymatic activities of PALs and VvUFGT were significantly higher in LT thermal regime. This was consistent with the transcriptomic data for PALs, whereas clearly discordant with the VvUFGT expression profile. These enzymatic assays clearly support the higher levels of anthocyanins and flavonols observed in berry skins under LT conditions, and that a post-transcriptional mechanism may be crucial in the regulation of the late steps of anthocyanin biosynthesis. Recent researches have revealed that post-translational mechanisms may play significant roles in the regulation of phenylpropanoid/flavonoid metabolic pathways. In Arabidopsis and apple (*Malus domestica*), where MYB transcriptions factors are required for anthocyanin accumulation and for the expression of structural genes in the anthocyanin biosynthesis pathway, it has been demonstrated that the repression of anthocyanin accumulation in darkness requires an interaction between a proteasome protein complex and MYBs (Li et al., 2012; Maier et al., 2013). Furthermore, a post-translational regulation mediated by the ubiquitin/26S proteasome system of the anthocyanin-related TRANSPARENT TESTA 8 (TT8) transcription factor has been detected in Arabidopsis (Patra et al., 2013). The possibility of a post-transcriptional regulation of the enzymatic steps of the pathway has also been described, showing that a multiple-level control governs the enzymatic activity of PAL (Zhang et al., 2013; Zhang and Liu, 2015).

The analysis of peroxidase activity revealed a significant increase under HT conditions, supporting that this class of enzymes may trigger the temperature-dependent degradation of anthocyanins (Mori et al., 2007). Our transcriptomic survey did not reveal a significant increase in the gene expression of the recently characterized VvPrx31 (Movahed et al., 2016) in HT conditions compared to LT, suggesting that new peroxidases isoforms (as peroxidase 50) may be involved in anthocyanins degradation following increasing temperature.

Overall, it is possible to hypothesize that different environmental conditions could influence anthocyanin biosynthesis and degradation in grapevine through post-transcriptional modifications of key structural genes of the pathway, such as UFGT and peroxidases.

## Conclusion

Our results provide valuable insights into the understanding of the mechanisms that underlie Sangiovese berries response to changes in growing temperatures that could be useful to identify the most suitable areas for Sangiovese cultivation under current climate change, given the great sensitivity of this cultivar to the increasing temperature. Two thermal regimes characterized by difference of 5°C in average and 7°C in maximum air temperature, specifically affected berry ripening. The LT regime delayed ripening in the early phases and had great impact on grape phenolic composition and on the activity of some enzymes involved in flavonoid biosynthesis, enhancing the accumulation of anthocyanins and flavonols. Conversely, berries grown at HT showed an increase in peroxidase activity, which could concur to the reduced accumulation of flavonoids found in these conditions. Transcriptional analyses identified the existence of a strong effect of both thermal regimes on the whole transcriptome, but the partial correlation between biochemical and molecular data requires further research to elucidate the existence of post-transcriptional and post-translational mechanisms involved in the balance between biosynthesis and degradation of flavonoids and in particular of anthocyanins.

## Author Contributions

CP performed the RNA extraction, contributed to the enzymatic and HPLC analyses and wrote the manuscript. SDS conducted the microarray experiments, interpreted the microarray data and wrote the manuscript. SZ and GT designed the microarray experiments and critically revised the manuscript. NM, GA, and GV sampled the material and contributed to the enzymatic and HPLC analysis. IF conceived and supervised the study, wrote and critically revised the manuscript.

## Conflict of Interest Statement

The authors declare that the research was conducted in the absence of any commercial or financial relationships that could be construed as a potential conflict of interest.

## Footnotes

1 mev.tm4.org/

2 http://www.ncbi.nlm.nih.gov/geo/query/acc.cgi?acc=GSE92864
