# Supplementary figures and images for: Whole Plant Temperature Manipulation Affects Flavonoid Metabolism and the Transcriptome of Grapevine Berries
Source: Front Plant Sci. 2017 Jun 6;8:929. doi: 10.3389/fpls.2017.00929 (PMC5460295; doi:10.3389/fpls.2017.00929)

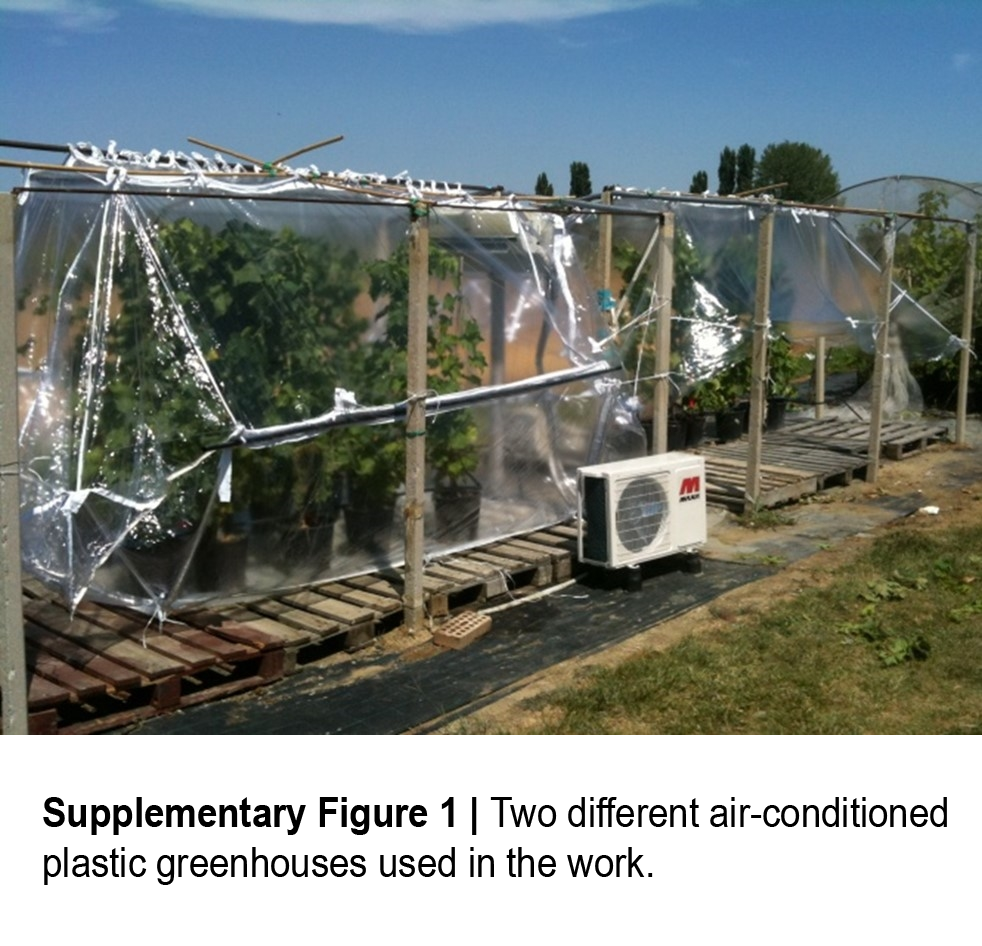

Supplement: Supplementary file 5 [file Image_1.TIF]

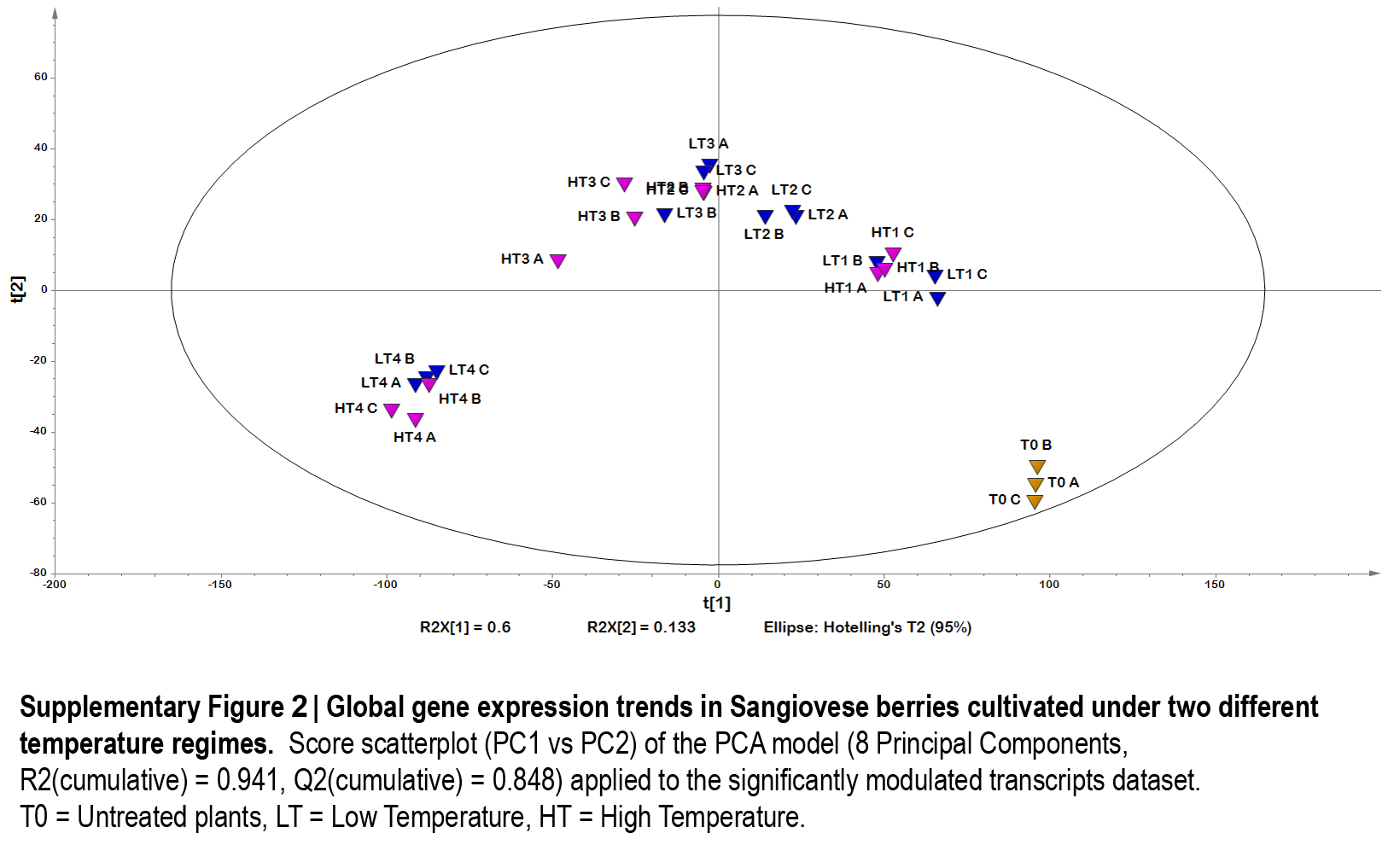

Supplement: Supplementary file 6 [file Image_2.TIF]

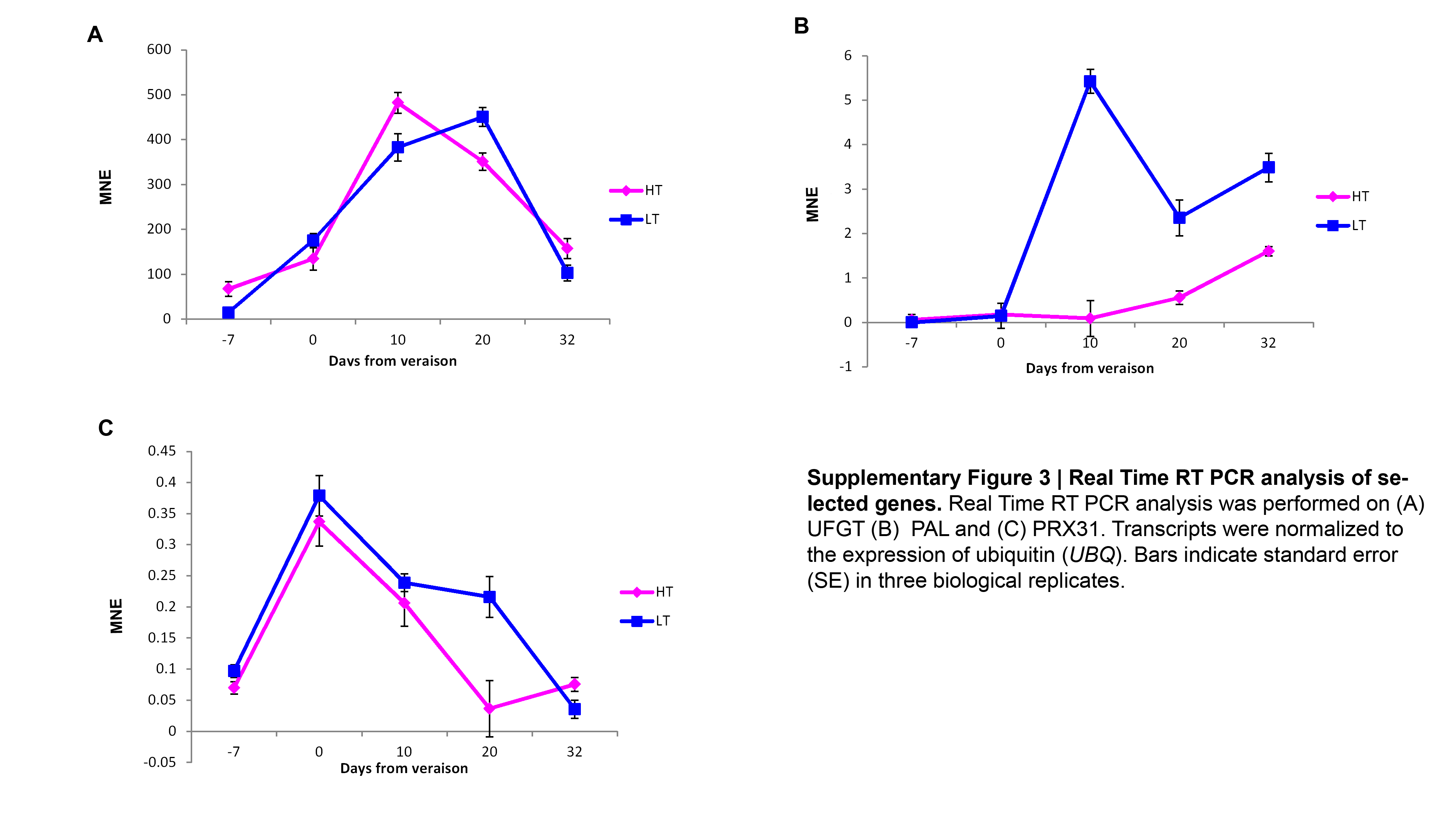

Supplement: Supplementary file 7 [file Image_3.TIF]

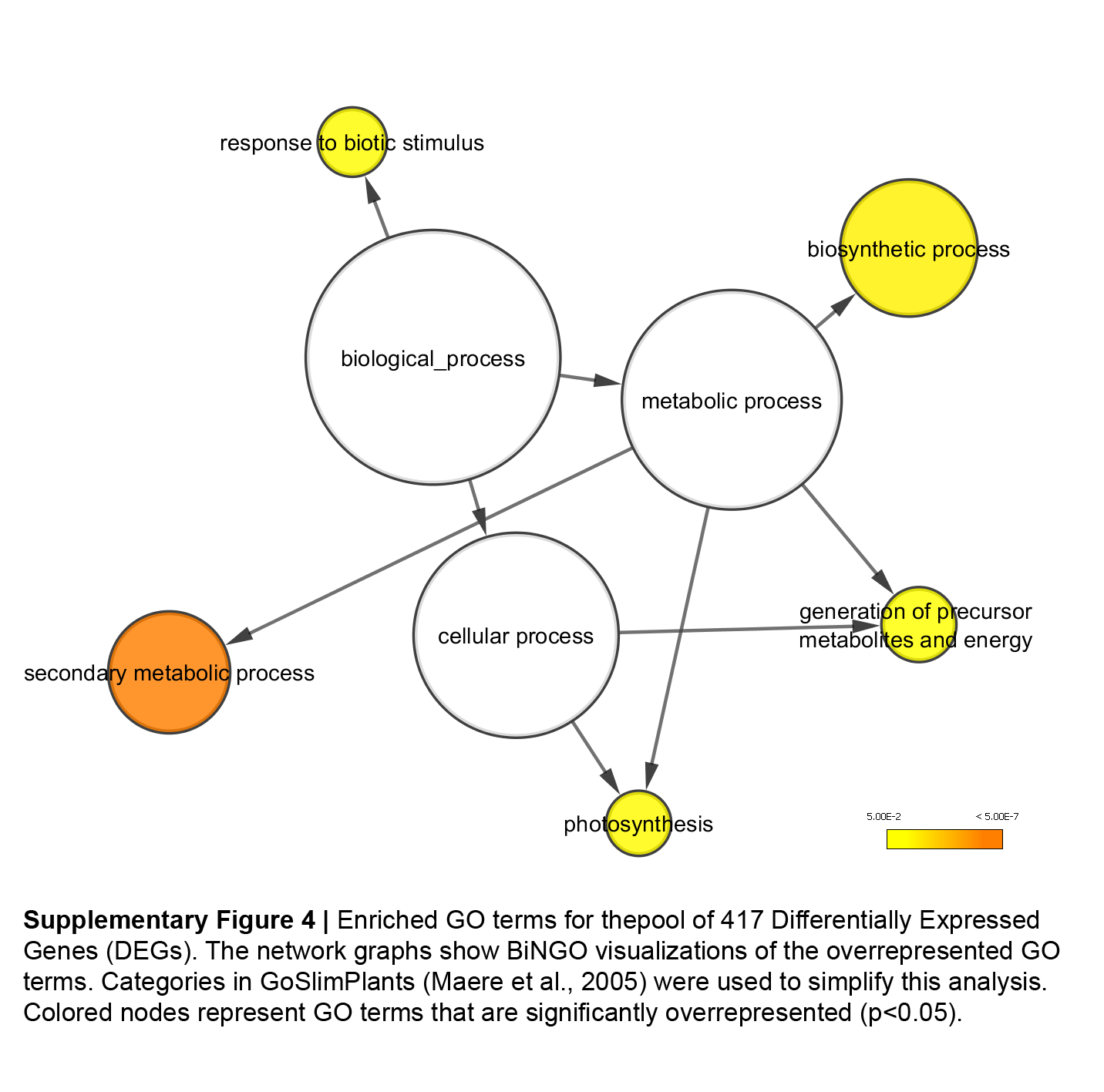

Supplement: Supplementary file 8 [file Image_4.TIF]

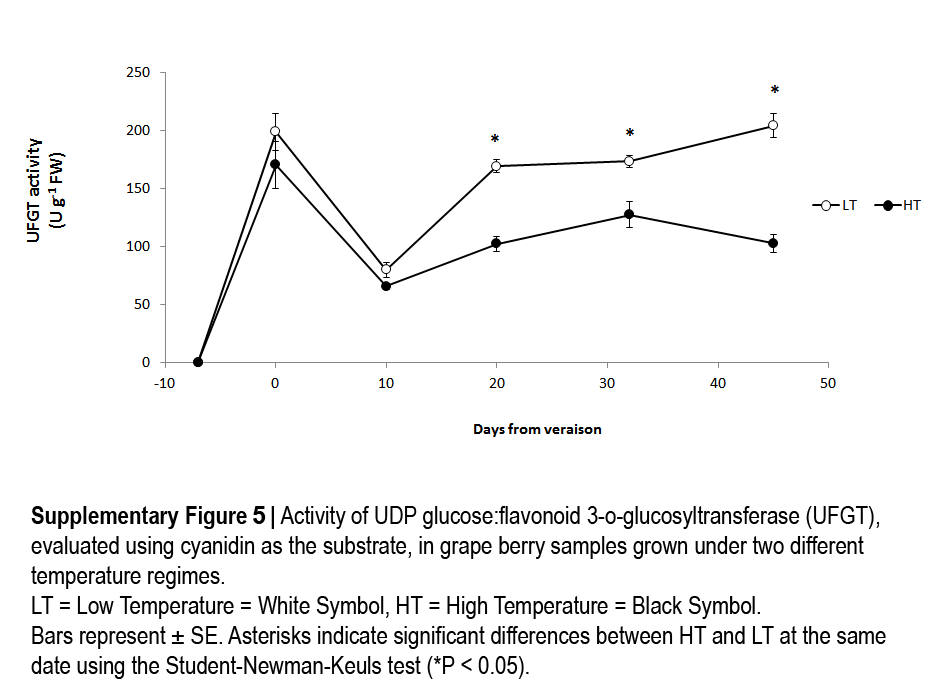

Supplement: Supplementary file 9 [file Image_5.TIF]
